# Design of Helical Capacitance Sensor for Holdup Measurement in Two-Phase Stratified Flow: A Sinusoidal Function Approach

**DOI:** 10.3390/s16071032

**Published:** 2016-07-04

**Authors:** Lam Ghai Lim, William K. S. Pao, Nor Hisham Hamid, Tong Boon Tang

**Affiliations:** 1 Department of Electrical and Electronic Engineering, Universiti Teknologi PETRONAS, Bandar Seri Iskandar 32610, Malaysia; limlamghai@gmail.com (L.G.L.); hishmid@petronas.com.my (N.H.H.); 2 Department of Mechanical Engineering, Universiti Teknologi PETRONAS, Bandar Seri Iskandar 32610, Malaysia; william.paokings@petronas.com.my

**Keywords:** helical capacitance sensor, finite element method, two-phase flow, stratified flow, holdup measurement, sinusoidal

## Abstract

A 360° twisted helical capacitance sensor was developed for holdup measurement in horizontal two-phase stratified flow. Instead of suppressing nonlinear response, the sensor was optimized in such a way that a ‘sine-like’ function was displayed on top of the linear function. This concept of design had been implemented and verified in both software and hardware. A good agreement was achieved between the finite element model of proposed design and the approximation model (pure sinusoidal function), with a maximum difference of ±1.2%. In addition, the design parameters of the sensor were analysed and investigated. It was found that the error in symmetry of the sinusoidal function could be minimized by adjusting the pitch of helix. The experiments of air-water and oil-water stratified flows were carried out and validated the sinusoidal relationship with a maximum difference of ±1.2% and ±1.3% for the range of water holdup from 0.15 to 0.85. The proposed design concept therefore may pose a promising alternative for the optimization of capacitance sensor design.

## 1. Introduction

Horizontal two-phase flow occurs widely in the petroleum, nuclear, and chemical industries. Pipeline transportation of natural gas in the presence of a liquid phase or mixture of crude oil and water are examples of two-phase flow [1]. One of the most common observations in two-phase flow is the complete separation between the two phases at moderately low velocities, where such a phenomenon is known as stratified flow. On the other hands, bubbly, intermittent, and annular flows can be observed at higher velocities [2].

A number of techniques have been applied to measure the holdup in two-phase flow, e.g., X-ray, gamma ray, optical, ultrasonic, and capacitive method [3,4]. The definition of holdup can be found in [5]. Amongst all these, the capacitive method was often employed due to its relatively cheap cost, simple design, and non-invasive approach—one just needs to attach the electrodes on the outer surface of the nonconductive section of the pipe. Relatively high sensitivity to water content could be achieved in two-phase flow, owing to the disparity in their permittivity values [5,6,7,8,9]. The signals from the capacitance sensors had been studied to characterize and identify the flow patterns in horizontal two-phase flow [10,11,12]. In addition to two-phase flow measurements, the capacitive sensing technique has also been adopted in numerous applications, e.g., occupancy, motion, position, displacement, level, touch, pressure, humidity, and moisture detectors [13,14].

Finite element method (FEM) is one of the most widely used design and analysis tools for capacitance sensor. Several configurations of two-electrode capacitance sensors have been proposed and analysed, i.e., concave, helical, and ring structures. Although increasing the number of electrodes (typically eight or more) to create an electrical capacitance tomography (ECT) has been explored [15], such a technique involves solving an inverse problem that is rather challenging in nature. A brief review of holdup measurement in horizontal two-phase flow using two-electrode capacitance system is presented in Table 1. Note that there were also other groups who conducted similar experiments on vertical two-phase flow [7,8,16,17,18,19]. Xie et al. [20] and An et al. [21] used two-dimensional (2D) finite element models to investigate and optimize the sensitivity distribution of concave electrode for holdup measurement in different flow patterns. In order to reduce the dependency of angle orientation in concave design, Hammer et al. [22] proposed a helical shape electrode of 180° and 360°. This was further validated by Tollefsen and Hammer [23] using a three-dimensional (3D) finite element model, where helical design was found to be more robust against the variation of flow patterns, specifically in stratified flow.

As compared to a concave design of the same spatial resolution, Ahmed [25] suggested that ring design was more sensitive to void fraction measurement. Similarly, Reis and Cunha [29] conducted an experimental study on several configurations of capacitance sensors for holdup measurement in air-water smooth stratified flow. They reported that ring design was the best configuration due to the least dependency of air-water distribution, but also found that all designs showed some levels of nonlinear response. Jaworek and Krupa [31] further pointed out that the electric field was strongly localized in the gap separating the rings, which in turn causes the ring design to be less sensitive to the changes of holdup.

On the other hand, the design of the helical sensor could be optimized by adding guard electrodes to improve the homogeneity in the sensitivity distribution field of capacitance sensor [7,8,16]. Despite that, the nonlinearity in response still could not be eliminated completely due to the nonlinear behaviour of the electrostatic field [20,23]. Thus, De Kerpel et al. [28] proposed a flow pattern based calibration for capacitive void fraction sensor to counter the nonlinear response, albeit at the expense of computational cost. One of the major patterns observed for the holdup measurement in stratified flow was the nonlinear response assimilated to a sinusoidal function alongside the ideal response, and it was found in various sensor designs [23,27,28,29].

Instead of suppressing, we propose to exploit the sinusoidal response characteristics as a novel design concept of helical capacitance sensor for holdup measurement in two-phase stratified flow. Helical design is chosen because of its minimal dependency on angle orientation as compared to a concave design. At the same time, this is also due to its higher sensitivity as compared to a ring design [29]. The proposed design is simpler as guard electrodes are not required. We derive an approximation model of the sinusoidal relationship observed between the capacitance readings and the holdup values. Experimental studies based on air-water and oil-water two-phase stratified flows are carried out to validate the sinusoidal model. If modelled accurately, the approximation model can be used to calibrate the capacitance sensor to acquire the actual holdup for two-phase stratified flow.

## 2. Helical Capacitance Sensor

### 2.1. Conventional Design

Tollefsen and Hammer [23] demonstrated that 180° and 360° twisted helical sensors exhibited similar trends of response between the capacitance value and holdup, but at different measurement ranges. In this case, a larger capacitance value obtained from 360° helical configuration is preferable in order to increase the sensitivity of the sensor [20]. In addition, the pitch of helix is better correlated with the 360° helical configuration. Thus, the 360° twisted electrode is selected.

**Figure 1:**
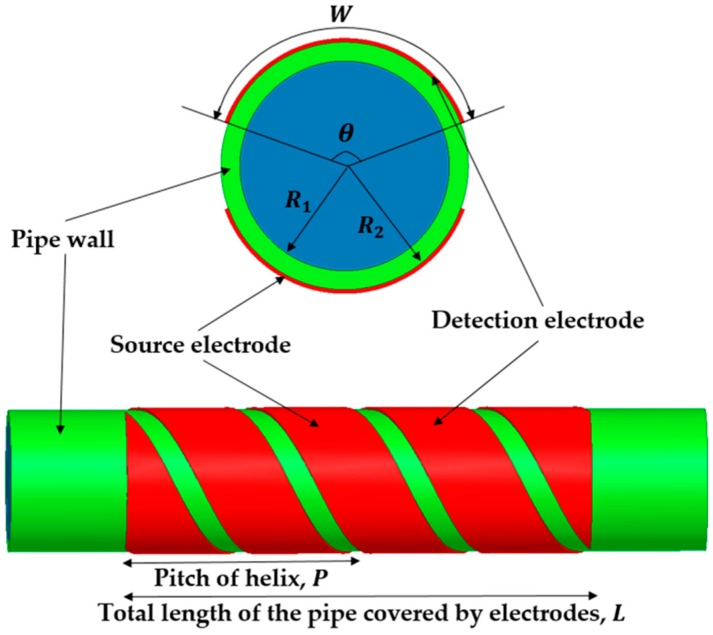
The structure of 360° twisted helical capacitance sensor design in 2D and 3D views.

Figure 1 shows the structure of the 360° twisted helical capacitance sensor in 2D and 3D views, which consists of source and detection electrodes. Parameters “W” and “θ” are the width and opening angle of source and detection electrodes, while “$R_{1}$” and “$R_{2}$” represent the inner radius and outer radius of the pipe. The thickness of the pipe wall is equivalent to $R_{2}-R_{1}$. The parameters W, θ, and $R_{2}$ are related by the following equation:

(1) $$W\;=\;\theta R_{2}$$

where the unit of θ is in radian. The pitch of helix “P” is defined as the length of one complete helix turn (360°), measured parallel to the axis of helix. In addition, the total length of the pipe covered by electrodes, denoted as “L”, is obtained by duplicating the number of complete helix turns and maintaining the pitch of the helix. This stage is crucial to minimize the fringe effect [20]. The relative permittivity of wall, gas, and liquid are represented as “$\varepsilon_{wall}$”, “$\varepsilon_{gas}$”, and “$\varepsilon_{liquid}$”, respectively.

### 2.2. Finite Element Model

FEM was used to design, analyse, and optimize the structure of helical capacitance sensor in 3D model. The voltage applied on the source electrode was 1 V and the detection electrode was 0 V. Due to the potential difference between the electrodes, the changes of the permittivity values in the measurement region can be observed through the capacitance value.

**Figure 2:**
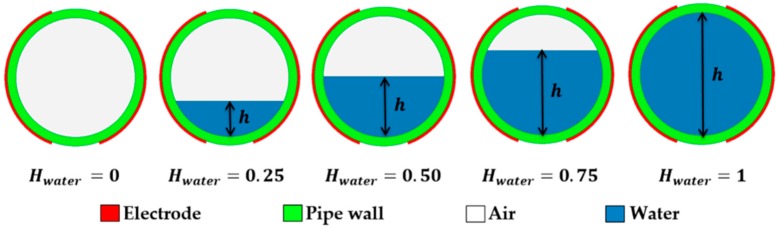
2D view of different holdup values for air-water smooth stratified flow.

Figure 2 displays 2D view of holdup values for air-water smooth stratified flow with different levels of water content, labelled as “h”. The water holdup is represented as “$H_{water}$”, where $H_{water}=0$ indicates the pipe is empty and $H_{water}=1$ indicates the pipe is filled completely with water. The static response of the 3D helical model was simulated to obtain the capacitance value of liquid holdups in stratified flow. By measuring the level of water content, the water holdup can be calculated as follows [32]:

(2) $$H_{water}=\{\begin{array}{ll} \frac{{cos}^{-1}\left(\frac{R_{1}-h}{R_{1}}\right)}{\pi }-\frac{\left(R_{1}-h\right)\sqrt{2R_{1}h-h^{2}}}{\pi R_{1}^{2}}, & h<R_{1}\\ 0.5, & h=R_{1}\\ 1-\frac{{cos}^{-1}\left(\frac{h-R_{1}}{R_{1}}\right)}{\pi }+\frac{\left(h-R_{1}\right)\sqrt{2R_{1}h-h^{2}}}{\pi R_{1}^{2}}, & h>R_{1} \end{array}$$

**Figure 3:**
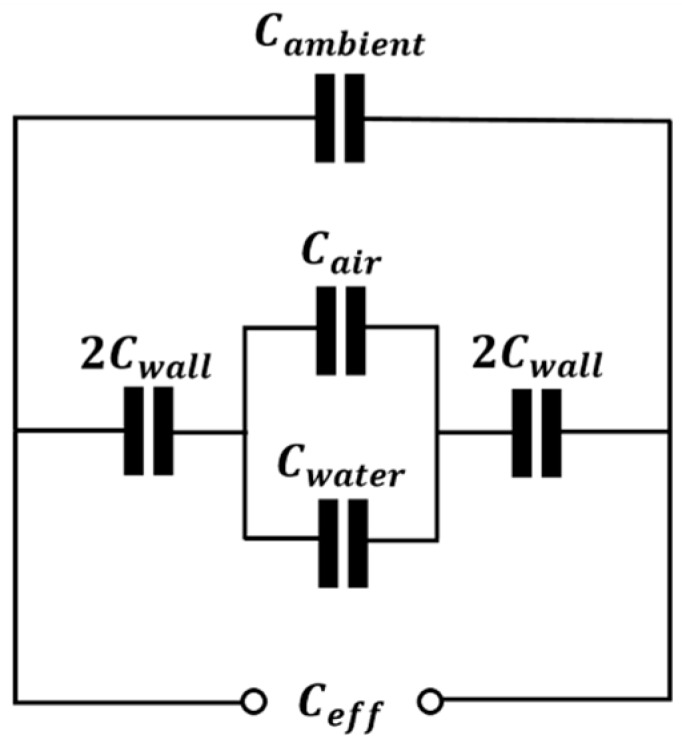
Equivalent circuit representation of helical capacitance sensor.

Figure 3 is an equivalent circuit representation of helical capacitance sensor displayed in Figure 2. In general, this would include the capacitance of ambient air, $C_{ambient}$ in parallel and the capacitance of pipe wall, in parallel and the capacitance of pipe wall, $C_{wall}$ in series with the capacitance of the two-phase components. However, note that $C_{ambient}$ and $C_{wall}$ are the constant parameters. The normalized capacitance, $C_{N,\;fem}$, obtained using FEM for air-water two-phase flow, can be defined as [28,29]:

(3) $$C_{N,\;fem}=\;\frac{C_{eff}-C_{\text{min}}}{C_{\text{max}}-C_{\text{min}}}$$

where $C_{eff}$ is the effective total capacitance comprised of air and water, and $C_{\text{min}}$ and $C_{\text{max}}$ are the minimum and maximum values of $C_{eff}$ when $H_{water}=0$ and $H_{water}=1$, respectively. In this context, the measurement range of the sensor is from $C_{\text{min}}$ to $C_{\text{max}}$ and the measurement span of the sensor is equal to $C_{\text{max}}-C_{\text{min}}$.

### 2.3. Approximation Model

An ideal capacitance sensor should display a linear relationship between $C_{N,\;fem}$ and $H_{water}$. However, when $H_{water}$ was less than 0.5, $C_{N,\;fem}$ was observed to be larger than the actual $H_{water}$ due to the closer proximity of the water phase with the electrodes as compared to the air phase. The only possible match between $C_{N,\;fem}$ and $H_{water}$ happened when the two phase components were uniformly separated in half. In contrast, when $H_{water}$ was larger than 0.5, $C_{N,\;fem}$ was lower than the actual $H_{water}$ due to the closer proximity of the air phase with the electrodes as compared to the water phase. As observed, the values of $C_{N,\;fem}$ were shifted from the ideal linear output and behaved identically to a sinusoidal function. A similar pattern was observed in simulation by other groups [23,27,28]. Since the nonlinear response cannot be eliminated absolutely, the sensor was optimized and designed in such a way that a sinusoidal relationship was generated between the capacitance reading and the holdup.

**Figure 4:**
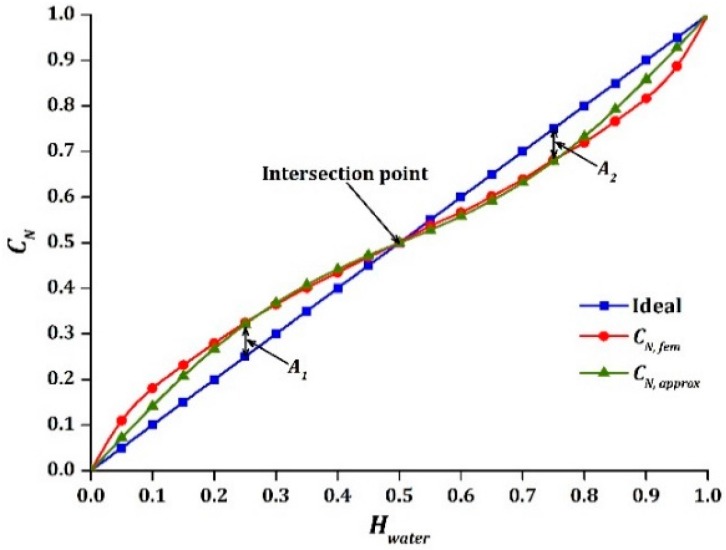
$C_{N,\;fem}$ and $C_{N,\;approx}$ versus $H_{water}$ .

The simulated design parameters of the helical capacitance sensor and the geometry of the pipe are presented in Table 2. As shown in Figure 4, the resulted output $C_{N,\;fem}$ was optimized to match the intersection point at $H_{water}=0.5$, closer to that of the ideal linear output. $C_{N,\;fem}$ also behaved as a symmetrical sinusoidal function throughout the entire range of $H_{water}$. The obtained results can be fit closely with the approximation model as below:

(4) $$C_{N,\;approx}\;=\;H_{water}+Asin\left(2\pi H_{water}\right)$$

where A is the amplitude of the sinusoidal function. In this case, the value of A was 0.071. The obtained value of A is calculated as follows:

(5) $$A\;=\;\frac{A_{1}\;+\;A_{2}}{2}$$

where $A_{1}$ and $A_{2}$ are the absolute difference between $C_{N,\;fem}$ and $H_{water}$ at $H_{water}=\;0.25$ and 0.75, respectively. The maximum absolute difference between $C_{N,\;fem}$ and $C_{N,\;approx}$ is displayed in Table 3, where a good agreement was achieved between 0.15 to 0.85 of water holdup. A slightly larger absolute difference was observed for water holdup of less than 0.15 and above 0.85, which needs to be further validated through experimental study. Importantly, the results showed that the sinusoidal output can be designed by modifying the pitch of the helix, regardless of other parameters.

## 3. Finite Element Analysis on Design Parameters

By simulating several different sets of design parameters and testing conditions, the effective total capacitance and their impacts on the response of sinusoidal output were examined.

### 3.1. Relative Permittivity of Two-Phase Components

**Figure 5:**
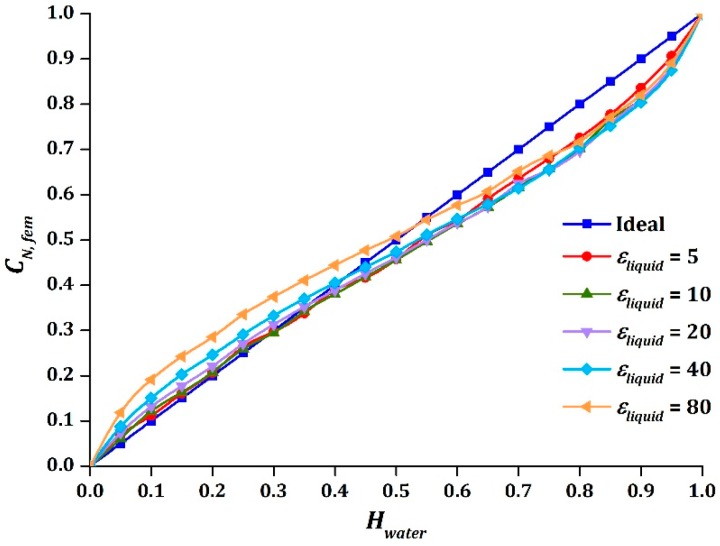
The effect on $C_{N,\;fem}$ due to variation of $\varepsilon_{liquid}$ .

Table 4 displays the capacitance values of different combinations of $\varepsilon_{gas}$ and $\varepsilon_{liquid}$. The values of $C_{\text{min}}$ were equal as the values of $\varepsilon_{gas}$ were kept constant, whereas the values of $C_{\text{max}}$ increased as the values of $\varepsilon_{liquid}$ increased. This indicated that the sensitivity of the sensor increases due to the significant difference in permittivity values of the two-phase components. As the values of $\varepsilon_{liquid}$ changed the values of $C_{\text{max}}$, the intersection point of sinusoidal output would be shifted, as displayed in Figure 5.

### 3.2. Geometry of Pipe

**Figure 6:**
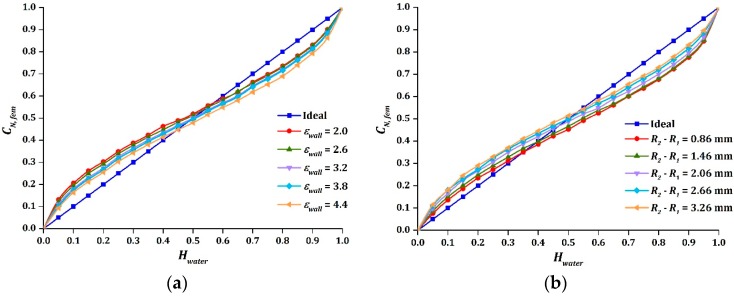
The effect on $C_{N,\;fem}$ due to variations of: (**a**) $\varepsilon_{wall}$; (**b**) $R_{2}-R_{1}$ .

The intersection point of sinusoidal output was observed to be shifted down, towards the direction of $H_{water}=0$, when the relative permittivity of the wall increases, as demonstrated in Figure 6a. In contrast, the intersection point moved in the opposite way, in the direction of $H_{water}=1$, when the thickness of the pipe wall increased, as shown in Figure 6b. The value of $C_{\text{max}}$ were severely affected by the thickness of the pipe wall, as shown in Table 5. This caused the measurement span of the sensor to be greatly reduced as the thickness increased. Thus, a thicker pipe wall made the sensor bulky and less sensitive to the changes of holdup [30].

**Figure 7:**
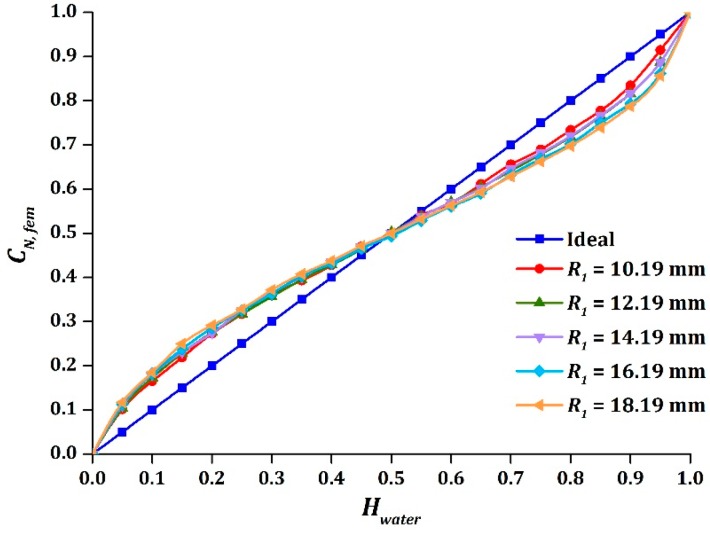
The effect on $C_{N,\;fem}$ due to variation of $R_{1}$ .

On the other hand, the amplitude of sinusoidal output was enlarged symmetrically as the inner radius of pipe increased, as displayed in Figure 7, while the intersection point was not affected. This indicated that the nonlinear response was more severe in larger pipes. The absolute difference of $\left|A_{1}-A_{2}\right|$ and the values of A for different sizes of pipe are summarized in Table 6, where it is seen clearly that A increased as $R_{1}$ increased and the small difference of $\left|A_{1}-A_{2}\right|$ indicated that the sinusoidal function was symmetrical.

### 3.3. Design Parameters of the Sensor

**Figure 8:**
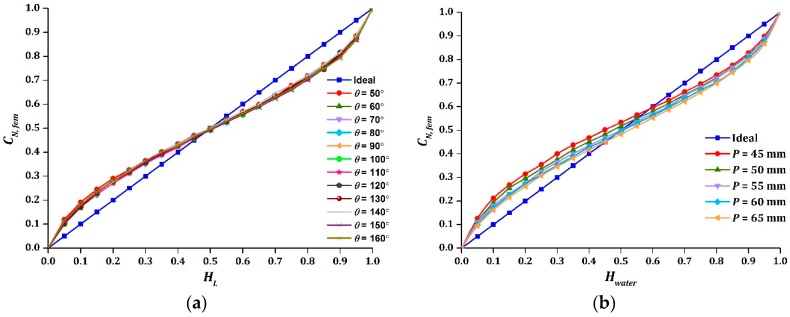
The effect on $C_{N,\;fem}$ due to variations of: (**a**) θ; (**b**) P .

The intersection point of sinusoidal output was found to be the same as the opening angles of electrode change, as shown in Figure 8a. However, the difference between $A_{1}$ and $A_{2}$ of the sinusoidal function for different values of θ increased, resulting in an asymmetrical response of the sinusoidal output. Besides, the measurement span of the sensor was strongly affected, which cannot be observed in Figure 8a. Hence, the absolute difference of $\left|A_{1}-A_{2}\right|$ and the measurement span for different θ are presented in Table 7. By comparing the absolute difference of $\left|A_{1}-A_{2}\right|$, an opening angle of 50° would yield the most symmetrical sinusoidal output, followed by an opening angle of 140°. In this case, the measurement span would be the next factor to consider when choosing the optimum θ. Theoretically, θ is related to the total surface area of the electrode, where greater θ would have a larger surface area and hence a bigger measurement span. In this case, a larger measurement span is desirable to improve the sensitivity of the sensor [20]. Thus, 140° was selected as the optimum θ.

The sinusoidal output needs to be symmetrical before applying the approximation model (Equation (4)), where the intersection point must lie closely to $H_{water}=0.5$ of that ideal line. Since the pitch of the helix can control the intersection point, as demonstrated in Figure 8b, a perfect sinusoidal function can be obtained by optimizing the pitch of the helix.

## 4. Experimental Setup

### 4.1. Capacitance Interface Circuit

**Figure 9:**
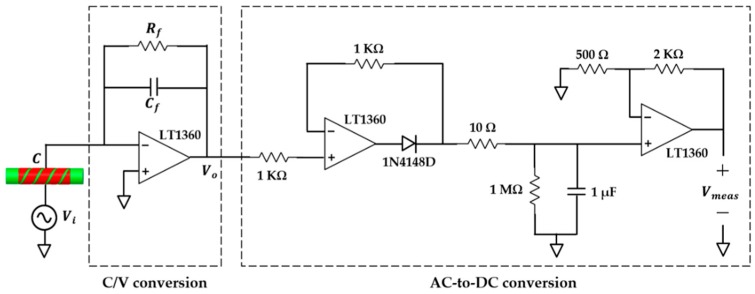
Schematic diagram of the capacitance interface circuit.

Figure 9 shows the schematic diagram of the capacitance interface circuit, which consists of capacitance-to-voltage (C/V) and AC-to-DC conversion circuits. The overall capacitance between the electrodes was in the range of 10 to 40 pF. A stray-immune and high signal-to-noise ratio measurement circuit is needed to maximize the accuracy and sensitivity of the system. Thus, an AC-based method [33,34] was employed for C/V conversion, where it was composed of an operational amplifier (LT1360) in inverting configuration mode. A resistor, $R_{f}$, in parallel with a capacitor, $C_{f}$, were chosen as the feedback impedance. In this experimental setup, $R_{f}=5\;MΩ$ and $C_{f}=22\;\text{pF}$.

A sinusoidal voltage of 1 V peak-to-peak generated by arbitrary function generator (AFG-3081) was used to excite the source electrode at frequency f=1 MHz, as suggested by other authors [19,29]. The detection electrode was connected to the virtual ground of the operational amplifier. Since the sensor was not covered by any external shield, any objects in close proximity with the sensor can cause significant interference to the capacitance reading. In our case, the surrounding of the sensor was not interfered with by any other physical object, except the ambient air, which has been considered in our model presented in Section 2.2. The capacitance value of the sensor, C, was linearly converted to sinusoidal output voltage, $V_{o}$.

The AC-to-DC conversion was comprised of active rectifier, peak detector, and an amplifier [35] for converting and amplifying the sinusoidal $V_{o}$ into DC value, $V_{meas}$. The final output, $V_{meas}$, was measured using a multimeter (GDM-8261A). A precision LCR meter (8110-G) with frequency of 1MHz and 1V AC was used to obtain the capacitance value of the sensor for linearity inspection between C and $V_{meas}$ for the same holdup values. The difference was found to be within ±0.1%, which indicated that the linearity error was within an acceptable range. The normalized output is denoted as “$V_{N,\;meas}$”, which has the same definition as $C_{N,\;fem}$.

### 4.2. Fabrication of Capacitance Sensors

**Figure 10:**
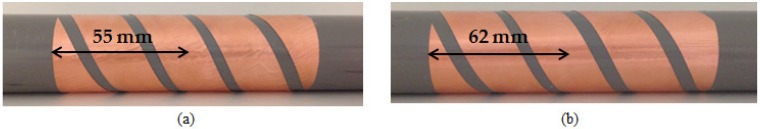
Helical capacitance sensor for: (**a**) Air-water; (**b**) Oil-water. Pitch size is shown for each design.

Figure 10 shows the actual photograph of helical capacitance sensors for air-water and oil-water stratified flow experiments, attached on the outer surface of the pipe. The electrodes were made of 0.075 mm thick copper foil coated with an electrically conductive acrylic adhesive surface. The pitch of the helix for air-water and oil-water experiments was determined to be 55 mm and 62 mm, respectively, from FEM simulation for achieving symmetrical sinusoidal outputs. In the design of sensors, the total complete turns of the helical sensor were doubled, where the total length of the pipe covered by electrodes were equal to 110 mm and 124 mm for air-water and oil-water experiments. This step was necessary to increase the measurement range of the sensor and subsequently minimize the fringe effect [20].

### 4.3. Static Two-Phase Stratified Flow Setup

**Figure 11:**
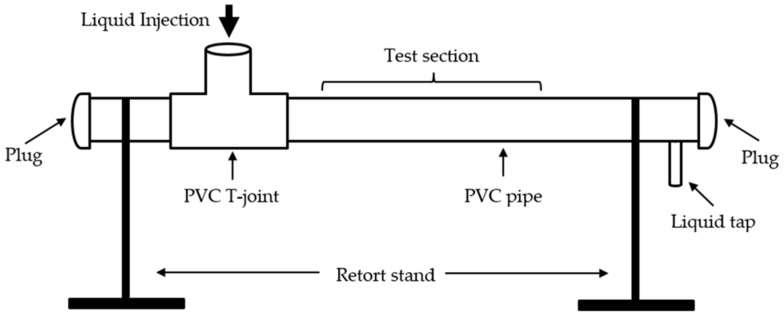
Schematic illustration of experimental setup for two-phase flow.

The schematic of experimental setup for two-phase flow is illustrated in Figure 11. It was composed of a pipe made of polyvinyl chloride (PVC), connected with PVC T-joint for liquid injection and sealed with a plug to prevent liquid leakage at both ends. The liquid tap was used to discharge the liquid inside the pipe and the retort stands were used to support the pipe horizontally at both ends. The capacitance sensor was attached on the test section of the pipe and the total volume of the pipe was measured. Due to the atmospheric pressure and density difference between the two-phase components, stratified flow can be observed easily inside the pipe as the less dense component (i.e., air or oil) would always float on the top of the denser component (i.e., water). Hence, the water holdup $H_{water}$ is equivalent to the ratio between the water poured into the pipe and the total volume of the pipe.

In this work, two static tests were conducted: air-water and oil-water stratified flow. Deionized water was used to prevent the interference of the resistive component. A small quantity of gasoline with density of 748 kg/m^3^ was used as the oil component. During the experiment, the temperature was kept at 25 ± 1 °C. The interface between the oil and water was allowed to settle down after each change in their volumes. The output voltage was recorded using the interface circuit, as discussed in Section 4.1. Table 8 lists the geometry of the pipe used for air-water and oil-water experiments.

## 5. Results and Discussion

### 5.1. Air-Water Stratified Flow

**Figure 12:**
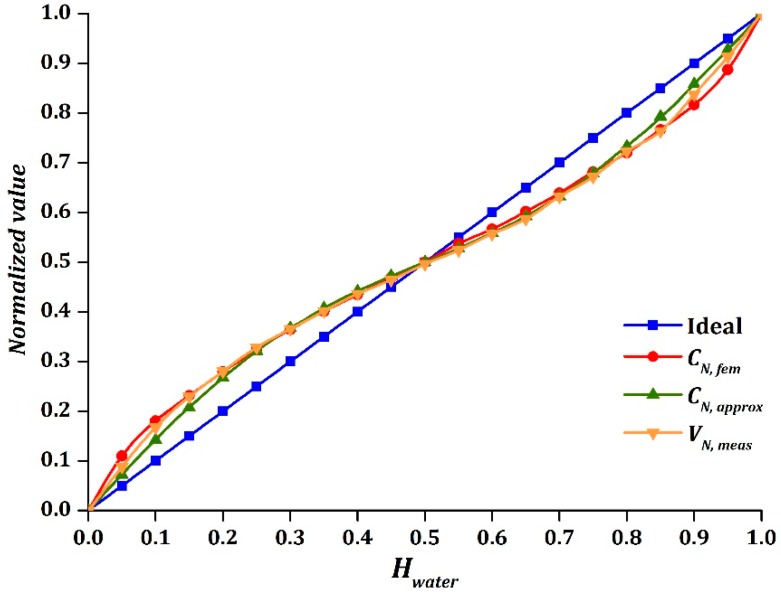
$C_{N,\;fem}$, $C_{N,\;approx}$, and $V_{N,\;meas}$ versus $H_{water}$ for air-water stratified flow.

Figure 12 shows the experimental results of air-water stratified flow. It was shown clearly that the output $V_{N,\;meas}$ behaved similar to a sinusoidal function, as observed in FEM simulation. The amplitude of the sinusoidal function was equal to 0.071, based on FEM calculation. The intersection point of $V_{N,\;meas}$ was closely located at $H_{water}=0.5$, indicates that the sinusoidal function was symmetrical. Table 9 tabulates the maximum absolute difference of $\left|C_{N,\;fem}-C_{N,\;approx}\right|$ and $\left|V_{N,\;meas}-C_{N,\;approx}\right|$ for air-water test. It was found that the experimental results had a higher accuracy as compared to the FEM simulation, particularly when water holdup was less than 0.15 and greater than 0.85. Note that smooth stratified flow was simulated using FEM. However, in reality, smooth stratified flow was hardly observed when the amount of water was either too little or almost full in the pipe due to the natural properties of water, which cause the distribution of the water in the pipe to be uneven. This could also be part of the reason why the range of water holdup $\left(0.126\leq H_{water}\leq 0.773\right)$ was limited in the experimental study of [29].

Two important properties that influence the experimental results are cohesion and adhesion of water. According to Marshall et al. [36], cohesion refers to the attraction of the same kind of molecules, where it holds hydrogen bonds together to create surface tension on water. On the other hand, adhesion refers to the molecular attractions at the interface of different kind of molecules. In this case, it has been observed that water molecules are inclined to stick to each other rather than to the inner surface of the pipe for water holdup of less than 0.15, which indicates that the cohesive force was stronger than the adhesive force. Since a trace amount of water was attached to the inner surface of the pipe, the capacitance readings would be expected to be lower than the simulated results in smooth stratified flow. As observed in Figure 12, $V_{N,\;meas}$ was smaller than $C_{N,\;fem}$ for water holdup of less than 0.15.

Meanwhile, the adhesive and cohesive forces of water were almost equal for values of water holdup from 0.15 to 0.85 as smooth stratified flow was easily formed inside the pipe. This showed that the simulation results and experimental results were close to each other. However, smooth stratified flow could not be generated as water holdup increased above 0.85. It was observed that the tendency of water to stick to the inner surface of the pipe was higher as adhesive force overwhelmed cohesive force. Due to this phenomenon, the capacitance readings would be expected to be higher than the simulated results in smooth stratified flow as more water attached to the inner surface of the pipe. Thus, the results showed that the values of $V_{N,\;meas}$ were larger than $C_{N,\;fem}$ for water holdup of greater than 0.85, where this brought the values closer to the approximation model. In addition, the air-water distribution had been re-simulated in FEM to validate the experimental result for water holdup of less than 0.15 and greater than 0.85. Overall, the output $V_{N,\;meas}$ obtained a good agreement with the approximation model, $C_{N,\;approx}$, where it was found to be even better than $C_{N,\;fem}$.

### 5.2. Oil-Water Stratified Flow

**Figure 13:**
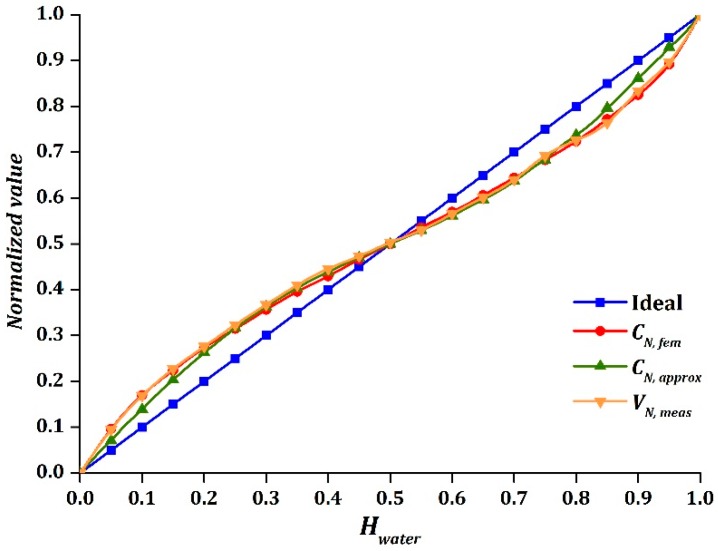
$C_{N,\;fem}$, $C_{N,\;approx}$, and $V_{N,\;meas}$ versus $H_{water}$ for oil-water stratified flow.

A clear and even separated interface between oil and water was observed after a few minutes for each change of holdup value. In addition, there were no cross-linking between oil and water when interfacial waves were absent, as reported by Al-Wahaibi and Angeli [37]. Thus, the flow pattern can be assumed to be smooth stratified. Figure 13 displays the experimental results of oil-water stratified flow. It was clearly shown that the output, $V_{N,\;meas}$, acted identically to a sinusoidal function, where the amplitude of the sinusoidal function was equal to 0.066. The sinusoidal function was proved to be symmetrical as the intersection point of $V_{N,\;meas}$ with the ideal line was nearly obtained at $H_{water}=0.5$. Table 10 presents the absolute difference of $\left|C_{N,\;fem}-C_{N,\;approx}\right|$ and $\left|V_{N,\;meas}-C_{N,\;approx}\right|$ of the oil-water test, where the experimental result was found to be very close to the simulation result.

It was also noted that the nonlinear response was greater when the permittivity difference between the two-phase components increased. As the value of amplitude implies the degree of nonlinear response of sinusoidal output, the air-water stratified flow was found to be shifted more significantly from the ideal response, as compared to the oil-water flow. This is manifested by the larger difference in permittivity values of air-water as compared to oil-water. Overall, the approximation model $C_{N,\;approx}$ obtained a good agreement in air-water and oil-water stratified flow for water holdup values from 0.15 to 0.85.

## 6. Conclusions

This paper reported a new and facile approach to the design and optimization of a helical capacitance sensor to measure the holdup of two-phase flow, specifically in a stratified pattern. The two phase components can either be gas and liquid or liquid and liquid. A sinusoidal relationship between the capacitance value and the holdup was observed and explored, where a good agreement was achieved between the FEM model and approximation model. In addition, all design parameters had been analysed and studied to determine their effects on the intersection point and symmetry of the sinusoidal function. The static experiments of stratified flow for air-water and oil-water further justified the proposed sinusoidal function with maximum differences of ±1.2% and ±1.3% for the range of water holdup from 0.15 to 0.85. In future, the flow loop test will be conducted to examine and investigate the performance of the sensor.

## Figures and Tables

**Table 1:** A brief review of holdup measurement in horizontal two-phase flow using two-electrode capacitance system.

Authors	Electrode Design	Guard Electrodes	Inner Diameter of Pipe	Two-Phase Components
Geraest and Borst [24]	Helical	Yes	5 mm and 50 mm	Air-water
Tollefsen and Hammer [23]	Concave and helical	No	82 mm	Gas-oil, gas-water
Ahmed [25], Ahmed and Ismail [26]	Concave and ring	No	12.7 mm	Air-oil
Caniere et al. [27]	Concave	Yes	9 mm	Air-water
Demori et al. [9] and Strazza et al. [5]	Concave	Yes	21 mm	Oil-water
De Kerpel et al. [28]	Concave	Yes	8 mm	Vapour-liquid
dos Reis and da Silva Cunha [29]	Concave, helical, and ring	No	33.85 mm	Air-water
An et al. [21]	Concave	Yes	10 mm	Oil-water
Zhai et al. [30]	Helical	Yes	20 mm	Oil-water
This paper	Helical	No	28.38 mm	Air-water, oil-water

**Table 2:** Design parameters of helical capacitance sensor and geometry of pipe.

Parameters	Values
$R_{1}$	14.19 mm
$R_{2}$	16.85 mm
P	55 mm
L	110 mm
θ	140°
$\varepsilon_{wall}$	3.2
$\varepsilon_{gas}$	1
$\varepsilon_{liquid}$	80

**Table 3:** Maximum absolute difference between $C_{N,\;fem}$ and $C_{N,\;approx}$ .

Water Holdup	Maximum Absolute Difference (%)
$H_{water}\leq 0.15$	3.9
$0.15\;<\;H_{water}<0.85$	1.2
$H_{water}\geq 0.85$	4.2

**Table 4:** Capacitance values of different combination of $\varepsilon_{gas}$ and $\varepsilon_{liquid}$ .

$\varepsilon_{gas}$	$\varepsilon_{liquid}$	$C_{min}$ (pF)	$C_{max}$ (pF)
1.0	5	5.987	10.521
1.0	10	5.987	13.945
1.0	20	5.987	17.516
1.0	40	5.987	20.606
1.0	80	5.987	22.476

**Table 5:** Capacitance values of different thicknesses of pipe wall.

$R_{2}-R_{1}$ (mm)	$C_{min}$ (pF)	$C_{max}$ (pF)	Measurement Span (pF)
0.86	3.960	47.714	43.754
1.46	4.751	34.322	29.571
2.06	5.495	26.866	21.371
2.66	5.987	22.476	16.489
3.26	6.553	19.800	13.247

**Table 6:** Absolute difference of $\left|A_{1}-A_{2}\right|$ and the values of A for different $R_{1}$ .

$R_{1}$ (mm)	$A_{1}$	$A_{2}$	Absolute Difference (%)	A
10.19	0.06669	0.06058	0.611	0.06364
12.19	0.06726	0.07125	0.399	0.06926
14.19	0.07441	0.06843	0.598	0.07142
16.19	0.07510	0.07958	0.448	0.07734
18.19	0.07847	0.08583	0.736	0.08215

**Table 7:** Absolute difference of $\left|A_{1}-A_{2}\right|$ and the measurement span for different θ .

θ (°)	$A_{1}$	$A_{2}$	Absolute Difference (%)	Measurement Span (pF)
50	0.07657	0.07174	0.482	7.587
60	0.07266	0.07952	0.686	8.687
70	0.06967	0.08064	1.098	9.965
80	0.06306	0.08420	2.115	11.102
90	0.06644	0.07787	1.143	12.179
100	0.06646	0.08246	1.600	13.199
110	0.06212	0.08526	2.314	14.174
120	0.06910	0.08143	1.233	14.645
130	0.06840	0.07671	0.831	15.626
140	0.07441	0.06843	0.598	16.489
150	0.06657	0.09035	2.378	17.272
160	0.07046	0.08755	1.709	17.856

**Table 8:** Geometry of the pipe.

Parameters	Values
$R_{1}$	14.19 mm
$R_{2}$	16.85 mm
$R_{2}-R_{1}$	2.66 mm
$\varepsilon_{wall}$	3.2

**Table 9:** Maximum absolute difference of $\left|C_{N,\;fem}-C_{N,\;approx}\right|$ and $\left|V_{N,\;meas}-C_{N,\;approx}\right|$ for air-water stratified flow.

Water Holdup	Maximum Absolute Difference (%)	
	$\left|C_{N,\;fem}-C_{N,\;approx}\right|$	$\left|V_{N,\;meas}-C_{N,\;approx}\right|$
$H_{water}\leq 0.15$	3.9	2.6
$0.15\;<\;H_{water}<0.85$	1.2	1.2
$H_{water}\geq 0.85$	4.2	2.8

**Table 10:** Maximum absolute difference of $\left|C_{N,\;fem}-C_{N,\;approx}\right|$ and $\left|V_{N,\;meas}-C_{N,\;approx}\right|$ for oil-water stratified flow.

Water Holdup	Maximum Absolute Difference (%)	
	$\left|C_{N,\;fem}-C_{N,\;approx}\right|$	$\left|V_{N,\;meas}-C_{N,\;approx}\right|$
$H_{water}\leq 0.15$	3.1	3.0
$0.15\;<\;H_{water}<0.85$	1.1	1.3
$H_{water}\geq 0.8$5	3.6	3.3

## Supplementary Materials

All relevant data are available from the database at the URL https://sites.google.com/site/medicalelectronicslab/research/data-deposition.
